# Liberation Medicine: Past, Present, and Future

**DOI:** 10.1007/s11013-025-09949-w

**Published:** 2025-10-16

**Authors:** Amand-Gabriel Führer, Julia Vorhölter

**Affiliations:** 1 Institute for Medical Epidemiology, Biometrics and Informatics, Martin Luther University Halle-Wittenberg, Halle (Saale), Germany; 2 Department ‘Anthropology of Politics and Governance’, Max Planck Institute for Social Anthropology, Halle (Saale), Germany

**Keywords:** Politically engaged medicine, Structural competency, Bodily rebellion, Concrete utopia

## Abstract

In her book *Death Without Weeping* (1992), Nancy Scheper-Hughes coined the term “liberation medicine,” which aims to place the individual experience of illness in a larger social context and use it as a starting point for critical thinking and resistance. Illness, so the basic premise of liberation medicine, is a form of resistance that can be turned into an effective political strategy. Accordingly, medicine is understood to have the potential for a “critical practice of freedom” that can create spaces for patients and medical staff in which new ways of dealing with human suffering are negotiated. Taking Scheper-Hughes’s reflections as a starting point, this editorial introduction to the special section conceptually develops the notion of liberation medicine, outlines how it relates to similar concepts and debates, and sketches what it might mean in the contemporary era. We argue that radically rethinking health and health care is a powerful way to rethink, and change, society at large. In this sense, we understand liberation medicine, following Wilder (2022), as a “concrete utopia.”

Medicine, the hospital, and the clinic … can be isolated, closed off, from the external world and from the experiential world of patients. Or they can provide a space where new ways of addressing and responding to human misery are worked out.… [What might medicine become] if … it could see the suffering that enters the clinic as an expression of the tragic experience of the world [?] We might have the basis for a liberation medicine, a new medicine, like a new theology, fashioned out of hope. (Scheper-Hughes, 1992: 215)

In her book *Death Without Weeping* (1992), Nancy Scheper-Hughes coined the term “liberation medicine,” which aims to place the individual experience of illness in a larger social context and use it as a starting point for critical thinking and resistance. Illness, so the basic premise of liberation medicine, is a form of resistance that can be turned into an effective political strategy. Accordingly, medicine is understood to have the potential for a “critical practice of freedom” that can create spaces for patients and medical staff in which new ways of dealing with human suffering are negotiated. Although she does not fully develop the idea, Scheper-Hughes speculates about the potential and, in fact, the moral obligation of medicine. She imagines a medicine that not only treats illness itself, but also reflects, and if possible acts, on the structural inequalities that cause illness.

Taking Scheper-Hughes’s reflections as a starting point, this Special Section aims to conceptually develop the notion of liberation medicine and what it might mean in the contemporary era. To this end, the different contributions focus on the following questions: How do marginalized bodies, individuals, and communities maneuver medical landscapes, and what would happen if their illnesses were seen not as weaknesses but as acts of bodily rebellion or starting points for politicization and new solidarities (Dasgupta, 2025 Pinto, 2025; Scheper-Hughes, 2025)? What are the opportunities, and where are the limits of a politically engaged medicine, today and in the past (Mair, forthcoming)? To what extent is it possible for doctors and other medical actors not only to look at individual bodies in treatment but also to reflect on and question the structures that (co-)determine illness? What would this entail for medical practice and training (Holmes; Bourgois, forthcoming)? And what opportunities for broader social change would arise if the current system shifted from evidence-based medicine to a context- or even solidarity-based medicine (Aragon Martin, 2025)?

Hereby, the authors approach the question of liberation medicine from various angles and perspectives: that of the doctor-anthropologist (Aragon Martin) and the researcher-doctor-activist (Mair), who reflect on the problems they have encountered in their clinical and activist work and use those as starting points for thinking about alternative medical spaces; that of marginalized communities in Delhi, who write about their experiences of alienation and neglect, but also care and solidarity in a large hospital (Dasgupta); and that of rebel bodies (Scheper-Hughes) and political protestors who seek recognition—and not simply a cure—for their pain (Jacob Pinto). Together with the introduction and two commentaries, the various contributions use liberation medicine as a lens to reflect on the radical potential of pain, illness, and of the medical system itself.

## Historical Legacies: From Liberation Theology to Liberation Medicine

As the commentaries by Philippe Bourgois and Seth Holmes (forthcoming) explore in more detail, Scheper-Hughes’s idea of a liberation medicine draws on a rich legacy. It builds on earlier concepts and approaches—especially liberation theology (Gutierrez, 2023; see also Farmer, 2003), but also liberation psychology (developed by Martín-Baró, see Aron & Corne, 1996), or liberation philosophy (Dussel, 1985)—which were developed in other disciplines to rethink the causes of inequality and oppression and find new, potentially radical ways of addressing them.

Rooted in Marxist and anti-colonial thought, these “liberatory approaches” seek to challenge the status quo by fighting dominant ideologies (especially capitalism, imperialism, classism, Whiteness) and related power structures. Furthermore, liberatory approaches read individual suffering and illness as potential symptoms of systemic failure and inequity, or—in Scheper-Hughes’s terms (2025)—as a bodily rebellion and a form of refusal to live under or endure oppressive conditions. Consequently, liberatory approaches prioritize critical praxis over abstract academic knowledge or principles, and rather than being satisfied with diagnosing and treating individual symptoms, they aim for large-scale social change. Most importantly, perhaps, liberatory approaches argue that genuine change requires the equal participation of, or even leadership by, those people and communities who are marginalized in the current system. Liberation medicine then, if understood as part of this tradition, is first and foremost a bottom-up approach that seeks to “elicit the experiences and views of poor people and to incorporate these views into all observations, judgements and actions” (Farmer, 2003: p. 146). Similar to Freire’s (2000 [1968]) proposition for a “pedagogy of the oppressed,” which aims to radically rethink the relationship between teacher, student and society at large, liberation, medicine seeks to challenge and change conventional power dynamics within the medical system (between medical practitioners and patients, but also between different care workers, see Nelson, 2011; Mukhopadhyay, 2016). It builds on the assumption that both illness and care can be starting points for reflection, recognition, politicization, and solidarity, and thus for new political imaginations^1^ (Abadía-Barrero, 2022).

Despite the long history of references to “liberation” in fields traditionally concerned with other goals, the potential of liberation *medicine*—as an analytical concept and as an ethical imperative for practice—has been little explored in anthropological and medical literature.^2^ Some authors (e.g., Dubal, 2018; Holmes, 2013; Mukhopadhyay, 2016) have evoked the term when thinking about ways of overcoming the shortcomings and harms of current medical systems, but there is, as of yet, no coherent framework or theorization. Therefore, this editorial introduction outlines a general idea of what liberation medicine could mean and how it relates to similar concepts and debates. Our aims in proposing the concept are twofold: First, we believe liberation medicine is a useful ‘uniting banner’ to curate a conversation between different disciplines and fields of practice which aim to radically reform, or revolutionize, the current (bio)medical system.^3^ Second, we use the concept to reflect on, and take seriously, the idea that radically rethinking health and health care is a powerful way to rethink, and change, society at large. In this sense, we understand liberation medicine, following Wilder (2022), as a “concrete utopia” .

## Liberation Medicine as Uniting Banner

Many ideas underlying the concept of liberation medicine are not radically new but have been developed, to different degrees, in a range of ‘medicine-critical’ disciplines and fields of practice, including medical anthropology, social medicine, public health, and health care activism. Our aim here is to bring these dispersed debates together and sharpen them under a new lens, with the idea of making them accessible—and interesting—to a new generation of scholars and especially practitioners.

Over the years, liberation-medicine-related ideas have been debated under different banners ranging from antipsychiatry (Goffman, 1961; Laing, 1967; Szasz, 1961) to anti-colonial medicine (e.g., Fanon, 2004, but see also recent debates on anti-colonial / ‘decolonized’ global health^4^). Some of the epistemic questions, especially concerning the ultimate causes of illness and the political economy of health, have been raised in discussions around the social determinants of health (Wilkinson & Marmot, 2003; Krieger, 2011; see also Virchow, 1985), and—more critically—in Marxism-inspired debates on medicine under capitalism (Engels, 1969 [1845]; Navarro, 1983; or, more recently, Lewis, 2019, 2022; Breilh, 2021). Based on these and other critical analyses, different approaches have been developed to change mainstream medical practice, for instance, by reforming and politicizing medical training through structural competency programs (Hansen & Metzl, 2019; Neff et al., 2020; Piñones-Rivera et al., 2024) or changing research practices (Rasheed, 2021).

All radical critiques share the assumption that mainstream medicine originates in and upholds classist, racialized, and imperial power structures, and that it does not serve the interests of, and in fact often harms, the most marginalized members of society. Consequently, they all grapple with the question of whether, and how, it is possible to reform the medical system—or whether such endeavors should be abandoned in favor of building “abolitionist” (Harney & Moten, 2013) or “fugitive” (van der Waal, 2024) health care.

Below, we group the different imaginations of and pathways toward a liberation medicine along this spectrum. Ideas and concrete attempts range from those that seek to re-appropriate and restructure health care institutions to those that seek to expand “the undercommons of autonomous networks” (van der Waal, 2024), to those that use medicine-related issues as starting point to lobby for socio-political change beyond the clinic.

Different approaches come with different challenges and risks. While ideas and attempts to reform the medical system are often quickly depoliticized, deradicalized, and coopted (Scheper-Hughes, 2025), complete ‘de-medicalization’ often leaves vulnerable individuals without any access to proper care—as the history of deinstitutionalizing mental health care in the US has powerfully demonstrated (Braslow & Messac, 2018; Estroff, 1985). Furthermore, while forging political identities out of what mainstream society, including some of those affected, read or experience as illness or disability—as promoted, for instance, by crip or mad studies (Kafer, 2013; LeFrançois et al., 2013)—can be empowering, it also comes with the risk that “disability” [or madness] becomes “another (unmarked and unnuanced) category to add to a laundry list of identities subsumed under diversity” (Friedner & Weingarten, 2019: p. 488).

By bringing together disparate ideas, disciplines, and debates under the banner of liberation medicine, we hope to generate a new conversation between fields that often exist in parallel. While it is ‘good to think with’ as an analytical concept for academic analysis, the term ‘liberation medicine’ also seems to evoke curiosity among critically minded clinicians—something we experienced when we organized the workshop on which this Special Section is based.^5^ Liberation medicine, then, as we imagine it, bridges theoretical reasoning, engaged research, activism, and clinical practice with the aim of instigating systemic changes, not just in the medical system but in society at large. However, liberation medicine is not prescriptive and does not offer ready-made ‘toolkits’ for reform. Rather, it is a decentralized, bottom-up, experimental, and utopian approach that starts from the assumption that bodily experiences like pain or illness, and related positive or negative encounters with medical care, can be eye-opening and transformative, for patients and practitioners alike.

## Liberation Medicine as Concrete Utopia

Our attempts to theorize liberation medicine start from the assumption that pain and illness are powerful experiences and that medicine is a powerful discipline. We contend that medicine is always political, and that medical actors—like theologians during the heyday of liberation theology—not only have an important voice in society but also witness (at times unconsciously) social injustice and its related harms on a daily basis (Farmer, 2001; Fanon, 2004 [1963]). Liberation medicine, therefore, involves both social analysis and critical praxis. It can be both an analytical tool for reflecting on existing ideas and initiatives and a vision for societal transformation, or what Gary Wilder (2022) calls a “concrete utopia.” Arguing against widespread political pessimism, fatalism, and ‘realism’ that dismiss attempts to imagine a different future and society, he writes:

[T]he opposite of political pessimism is not optimism. It is concrete utopianism. By utopian, I mean thought and action oriented toward that which appears to be, or is purported to be, impossible when such impossibility is only a function of existing arrangements. Concrete utopianism is not merely fanciful, phantasmatic, or speculative. It seeks to identify possibilities for alternative arrangements that may already dwell within, or be emerging from, the nonidentical order that actually exists. Such utopianism also seeks to identify concrete interventions that point beyond the logic and framework of the existing order. (Wilder, 2022: p. 9)

As a concrete utopia, liberation medicine emerges from the cracks of the existing system. Like liberation theology, it rests on the hope that a radically different future is possible and therefore transcends the pessimism, realism, and presentism that—according to Wilder (2022: p. 9)—characterize contemporary “Left” thinking and political theory in North America and Europa (see also Ferguson, 2010). In thinking with Wilder, we seek to identify glimpses of, and anchoring points for, a radically different form of medicine and, by extension, a radically different society.

Liberation medicine, as we understand it, operates at different levels and involves different actors: Starting with sick bodies and alternative ways of sense-making which challenge medical orthodoxy, it also requires physicians who are willing to refuse and unlearn conventional medical knowledge, who are willing to change their practice, and who are willing to ally with actors from outside the medical system to call for systemic change. While a fundamental restructuring of contemporary health care provision is an important first step, it is not a goal in itself but only a means to achieve social transformation. Citing concrete historical and contemporary examples as reference points, in the paragraphs below we speculate about what liberation medicine might look like.

## From ‘Rebel Body’ to ‘Rebel Patient’: Politicization through Illness

One starting point for liberation medicine is the individual body, or person, who experiences illness. The sick body, as Scheper-Hughes (2025) and many others (e.g., Sontag, 1978; Frank, 1995; see also Diedrich, 2007) have argued, is subversive and can function as a signifier of a sick system, as a “carrier of structure” (Dubal, 2018).

Illness experiences, while fundamentally challenging, offer opportunities for new insights (about one’s life circumstances, but also about the inadequacies or cruelties of the medical system) and subsequent politicization. Certainties relating to the body, the self, and one’s position in society and the world more broadly fall apart and can lead to “narrative wreckage” (Frank, 1995), leaving the sick individual not only alienated from his or her body but also isolated from the world and the other bodies that populate it. As Taussig outlines, this fragile moment is routinely used by biomedicine to reinforce capitalist notions of the body as a commodity, through which social relations that are implied in the body are disregarded and the body transformed into a thing (Taussig, 1980). As a result, the vulnerable moment of illness is used to reinforce the body as an object of investigation (for the physician) and as a tool (for the patient), instead of using it as a starting point for a process that brings to light, de-reifies, and liberates the potential “for dealing with antagonistic contradictions and breaking the chains of oppression” (Taussig, 1980).

### Different Pathways

In different ways, then, the ‘rebel body’ stands as a starting point for liberation medicine. Engaging illness as a refusal to comply could lead to the development of political consciousness—at an individual level, perhaps through the help of ‘rebel doctors’ and beyond, through coalescing with other sick bodies (Frank, 1995; Taussig, 1980).

Once illness is understood as more than bodily failure, different pathways for change become possible. On one end of the spectrum, individuals might find ways to transcend their former marginalized status in society: by becoming incorporated into the medical system as experts (as in contemporary peer support initiatives, e.g., Cubellis, 2018), as ‘traditional healers’ (as has been widely discussed in older medical anthropology literature, e.g., Silverman^1967^ or, more recently, Langwick, 2011), or when they are seen as possessed by, or in contact with, spirits (as in the classic case of the Zar-Bori ‘cults,’ see Lewis et al., 1991; for a more recent example, see, e.g., Turner, 2020).

On the other end of the spectrum, they may become activists against the existing (medical and social) system and either lobby for reform and the establishment of new care structures (e.g., patient-led AIDS and breast cancer activism that emerged in the 1980s in the US; see Diedrich, 2007), or radically withdraw from the system and its underlying worldview to create alternative networks (as was tried with the People’s Free Clinics run by the Black Panther Party, see Nelson, 2011), or alternative interdisciplinary health centers (Mair, forthcoming).

In this volume, Dasgupta (2025)  follows up on this perspective and shows how patients’ relatives over time turn into “veterans of the waiting room” and establish networks of mutual aid that fill the gaps left by the biomedical system. Similarly, Pinto (2025) elaborates on the networks of solidarity among people injured by French police during demonstrations and shows how their struggle for recognition—recognition that their pain is socially inflicted—could serve as a model for liberation medicine.

## The ‘Rebel Doctor’

Whether they are aware of it or not, doctors have enormous influence over how suffering is interpreted and addressed: They can, and often do, naturalize, depoliticize, or medicalize it. But they could also choose to reinterpret illness as systemic failure and ally with sick bodies or minds in “rebelling” (Scheper-Hughes, 2025) against exploitation, discrimination, and “willful blindness” (Bovensiepen & Pelkmans, 2020). Recent work in medical anthropology on the opioid crisis (e.g., Hansen et al., 2023) or gun/violence-related injuries (e.g., Ralph, 2014) shows just how powerful medical actors are in influencing how illness is socially interpreted and whether sufferers are seen as victims or criminals, who receives treatment (or sentencing) and what kind, and how medical interventions at every level have the chance to (re)create or contest social distinctions and structural violence.

Traditional approaches understand the physician’s role to be that of a gatekeeper who determines who is officially certified as sick and therefore temporarily exempted from their obligations, and who is deemed healthy enough to keep functioning (Parsons, 1951). In contrast, liberation medicine envisages another role for physicians: To understand illness as a legitimate expression of resistance (even though an expression that tends to imply a lack of other means of expression) and be willing to create a space in which this resistance can be translated, together with patients and social movements, into other, and more political, means of expression (see Dubal, 2018).

This by no means implies a romanticization of disease; liberation medicine would still treat diseases using the full range of biomedical interventions (Farmer, 2003)—but it would do so while carefully avoiding the introduction of reductionist etiologies that reify patients into objects (Taussig, 1980). Such liberatory care would aim to go beyond the immediate symptom and engage patients in a process that deciphers how “social relations are mapped into disease” (Taussig, 1980) and how social inequity is embodied in individual lives (Krieger, 2011, 2014).

To achieve this goal, it is not enough that health care providers embrace a liberatory epistemology and treat their patients in a structurally competent (Metzl & Hansen, 2014) way. In addition, health care provision needs to be restructured according to principles of solidarity rather than critical distance and authority, whereby medical practitioners would actively search out collaborations with non-medical partners to address structural influences on patients’ lives. Beyond the currently increasingly common inclusion of social workers in clinical teams, such collaborations could range from interdisciplinary diagnostic teams and the inclusion of patients in clinical routines (Horn, 1969; Kokkinidis & Checchi, 2021), to cooperating with communities (Seymour et al., 2018), to explicitly positioning clinical care within liberatory struggles (Frierson, 2020; Nelson, 2011; Sozialistisches Patientenkollektiv, 1979). In addition, as discussions with clinicians highlight, a liberatory medicine would need to develop new ways of allocating time that prioritize care.

### Different Pathways

As with rebel patients, liberation medicine can also start with changing doctors’ and other health care providers’ perception of illness and medicine: Instead of seeing illness as an individual event caused by biological aberrations—and medicine as a mechanism to act upon such events—they could start seeing illness as a form of resistance and medicine as an instrument in this process. This could be achieved, first and foremost, by radically reforming medical training (Holmes et al. 2011) so that future doctors or nurses would be better able to see and treat (if willing) “the suffering that enters the clinic as an expression of the tragic experience of the world” (Scheper-Hughes, 1992: p. 215). Beyond theoretical medical teaching, working in structures of health care provision that are explicitly situated within liberatory struggles (in protest camps, as part of social movements, or even in times of war) can have a more immediate politicizing effect. For instance, as Nelson’s (2011) analysis of the Black Panther Party’s Free People’s Clinics shows, working in such contexts can shape medical workers’ professional identities, help to develop resistant ethics, and also provide opportunities to learn structurally competent clinical practices. Including rotations in such settings into routine medical training could be another ingredient in the training of rebel doctors.

As with rebel patients, the politicization of medical workers could lead to various scenarios. On one end of the spectrum, medical workers might try to change their own practices and the systemic structures in which they operate (see, e.g., Farmer, 2003). For example, Mair (forthcoming) illustrates such a process using the example of health care activism in Germany and shows how the utopian potential of interdisciplinary clinics is curtailed by the hard realities of cost absorption and hierarchy within medical teams.

On the other end of the spectrum, health care workers might abandon the established system and try to set up alternative structures and networks, turning into ‘fugitive’ medical workers (van der Waal, 2024), or resorting to a ‘context-based medicine’ that aims to create a situated practice of medicine that prioritizes both integrating emotional and relational aspects as well as reflecting on power dynamics in clinical practice, as outlined by Aragon Martin (2025).

## Rebel Movements: Using Medical Issues to Lobby for Socio-political Change

In line with the premise that (certain)^6^ illnesses are an expression of oppression and social injustice, liberation medicine ultimately aims to address the social determination of disease by working toward social justice. To that end, it has to go beyond the effects of individual politicization—of patients and of doctors—and beyond changes in how medical care is organized and delivered. Instead, it should try to establish liberatory health care structures as focal points for social movements. Issues of health and disease tend to be “common concerns” (Xiang, 2022) for many people and across social boundaries (even though access to health care and healthy living is often highly unequal). Thus, mobilization around issues related to health and illness in certain neighborhoods, institutions, or populations can be a starting point for communal political action that sooner or later goes beyond medical issues and starts to question the unequal distribution of wealth, power, and access to societal resources.

### Different Pathways

Once again, liberation medicine at the level of social movements may have different starting points and follow different pathways. Some movements may emerge around specific health concerns and demands—historical examples include the HIV/AIDS-focused *Treatment Action Campaign* in South Africa (Forbath et al., 2010) and ACT-UP in the US (Gould, 2012), black protest movements in the US that mobilized around sickle cell anemia to raise consciousness about social inequality and their civil rights to health (Nelson, 2011; Wailoo, 2001), or the “Janes,” a network of women who provided abortions before it became legal in the US (van der Waal, 2024).

Conversely, social movements that have developed around other issues may discover that changes in medicine and health care are crucial to their cause—e.g., the climate change movement that now lobbies for planetary health (see, e.g., www.planetaryhealthalliance.org/planetary-health), or the initiatives by doctors and other health care workers to support Black Lives Matter (e.g., https://whitecoats4blacklives.org/; Leitch et al., 2021), or Latin American Social Medicine scholar-activists who incorporate decolonial approaches to support peace-building in Colombia (Laurens et al., 2023).

## Concluding Reflections

In his book, *A Labour of Liberation*, Baijayanta Mukhopadhyay (2016) writes: “[P]erhaps because we have made health such a technical topic, it has lost its political edge.” One of the key aims of liberation medicine, then, is to rediscover the political in medicine and to explore the conditions under which medicine could be liberating work.^7^

Throughout this Special Section, the articles shed light on different forms that the liberation of medicine could take. They use ethnography to highlight how individual pain and injuries can simultaneously mark marginalization and underpin the formation of new solidarities (Pinto, 2025; Scheper-Hughes, 2025), or how gaps in the medical system might be filled by patients and their relatives, fostering new social relations (Dasgupta, 2025). Examining more organized forms of rebellious care, they highlight how new ways of practicing medicine might emerge in the cracks of precarious care for marginalized groups (Aragon Martin, 2025) or how, once they start to take on defined roles within the health care system, new forms of care are themselves in danger of becoming institutionalized and coopted (Mair, forthcoming).

Thus, an overarching theme of this Special Section is the question of how an understanding of illness as rebellion against unbearable social conditions can be translated into practices of care, and how the latter, to be successful, needs to relate to established health care structures. Should liberation medicine restrict itself to ‘ephemeral places of care’ in the crevices of established health care structures? Should it infiltrate and attempt to reform those structures? Or should it leave the health care system behind and begin from scratch?

This, we hope, is open for experimentation.

## Data Availability

No datasets were generated or analyzed during the current study.

## Footnotes

1 In the conclusion to his book, Abadía-Barrero writes: “Medicine as political imagination is … not a purely rational or intellectual exercise of ideas. The way we politically imagine health is the result of a particular epistemology of care resulting from merged and socially constructed theories and praxes; it depends on people’s shared experiences, history, and materiality.… [T]he epistemology of care and medicine as political imagination are coproduced” (Abadía-Barrero, 2022: p. 227).

2 As we were finalizing this introduction, however, we noticed that the term ‘liberation medicine’ has recently been evoked by scholars (e.g., Abu-Sittah, 2025; Ge, 2025) to critique the role humanitarian medicine and global health have played in contexts of settler colonialism and occupation, in Gaza in particular. They argue that health care provision has always played a central role in the revolutionary struggle, but has become depoliticized and a “tool of imperial soft-power” (Abu-Sittah 2025) through the Global Health regime. In contrast to apolitical and imperial forms of humanitarian medicine, these authors call for a medicine in line with what Che Guevara termed “revolutionary medicine” (Guevara 1969). Differing somewhat from our understanding of liberation medicine—as outlined in this special section—revolutionary medicine seems to prioritize the active engagement of physicians in armed conflict over the analytical questions raised by understanding sickness as rebellion and the community-based action that hopefully follows from it.

3 When we speak of medical system in the singular, we refer to the hegemonic global health system that is governed by the WHO and other major health institutions, build around classificatory diagnostic regimes such as the ICD or DSM, and closely tied to the ‘medical industrial complex,’ i.e., a worldwide network of pharmaceutical, technological, and insurance companies, teaching and research institutions, and government organizations.

4 https://globalhealth.jhu.edu/anti-colonialism-in-global-health, accessed 11 September 2024.

5 The workshop emerged from a cooperation between the Max Planck Institute for Social Anthropology and the Institute for Medical Epidemiology, Biometrics and Informatics at Martin Luther University Halle-Wittenberg. It was held in Halle (Saale), Germany, in February 2024 and brought together medical students and practitioners, psychologists, anthropologists, public health scholars, and physician-anthropologists.

6 While it generally helps to ask if a given illness can be understood as an expression of resistance, it is also important to note that not all diseases stem from oppression (Scheper-Hughes, 2025).

7 For an inspiring reflection on this question see Berger (1967).
